# Electrochemical Sensor for Simple and Sensitive Determination of Hydroquinone in Water Samples Using Modified Glassy Carbon Electrode

**DOI:** 10.3390/biomedicines11071869

**Published:** 2023-06-30

**Authors:** Parisa Karami-Kolmoti, Hadi Beitollahi, Sina Modiri

**Affiliations:** 1 Department of Chemistry, Graduate University of Advanced Technology, Kerman 76311-33131, Iran; 2 Environment Department, Institute of Science and High Technology and Environmental Sciences, Graduate University of Advanced Technology, Kerman 76311-33131, Iran; 3 Polymer Department, Graduate University of Advanced Technology, Kerman 76311-33131, Iran

**Keywords:** electrochemical sensing, hydroquinone, voltammetry, modified electrode, MnO_2_ NRs/GO nanocomposite

## Abstract

This study addressed the use of manganese dioxide nanorods/graphene oxide nanocomposite (MnO_2_ NRs/GO) for modifying a glassy carbon electrode (GCE). The modified electrode (MnO_2_ NRs/GO/GCE) was used as an electrochemical sensor for the determination of hydroquinone (HQ) in water samples. Differential pulse voltammetry (DPV), cyclic voltammetry (CV), and chronoamperometry were used for more analysis of the HQ electrochemical behavior. Analyses revealed acceptable electrochemical functions with lower transfer resistance of electrons and greater conductivity of the MnO_2_ NRs/GO/GCE. The small peak-to-peak separation is an indication of a rapid electron transfer reaction. Therefore, this result is probably related to the effect of the MnO_2_ NRs/GO nanocomposite on the surface of GCE. In the concentration range of 0.5 μM to 300.0 μM with the detection limit as 0.012 μM, there was linear response between concentration of HQ and the current. The selectivity of the modified electrode was determined by detecting 50.0 μM of HQ in the presence of various interferent molecules. At the end, the results implied the acceptable outcome of the prepared electrode for determining HQ in the water samples.

## 1. Introduction

The existence of any form of life on earth is dependent on water, which is vital. Water contamination is the primary cause of the water crisis. Water contamination is primarily concentrated in areas of industrialization, urbanization, agricultural activities, etc. The level of water contamination is affected by the abundance of pollutants, their ecological impact, and the intended use of the water. Water contamination is a significant issue that has adverse effects on both the ecosystem and human health. Exposure to water contamination over an extended period is a leading factor in causing various health problems and diseases. Additionally, it can disrupt ecological environments, including plant and animal life, and affect food chains. Protecting water systems to maintain public health and detecting contamination are crucial factors in achieving water security [1,2].

Hydroquinone (HQ) (1,4-dihydroxybenze), as one of the major isomers of di-hydroxybenzene, has widespread application for preparation of photo-stabilizers, dyes, cosmetics, plasticizers, drugs, and pesticides. In fact, HQ has an extensive use in producing food additives, antioxidants, as well as hair dyes. However, since dihydroxybenze isomers have lower degradability and great toxicity in an ecological environment (according to the World Health Organization (WHO)), HQ is toxic to several aquatic organisms even at concentrations below 1 mg L^−1^ (9.1 µmol L^−1^) [3]. A main topic, which has been largely investigated in the environmental pollutants, is how to selectively detect dihydroxybenzene [4,5]. Hence, experts in the field have provided several techniques of HQ detection such as surface-enhanced Raman spectroscopy [6], spectrophotometry [7], capillary electrophoresis [8], chemiluminescence [9], calorimetric sensor [10], gas chromatography-mass spectrometry [11], and high-performance liquid chromatography [12]. However, a number of the mentioned techniques suffer from complex operations, costly equipment, hard separation procedure, and high toxic organic solvents, but since HQ has an electroactivity property, electrochemical approaches could be used for its detection [13]. Hence, the development of simple and sensitive electrochemical sensors for the determination of HQ has received considerable attention [14,15,16,17,18,19,20,21,22].

Electrochemical methods have been increasingly attractive for features such as faster responses, simplified operations, higher sensitivity, good selectivity, insignificant content of the samples, and inexpensiveness [23,24,25,26,27,28,29,30]. Nonetheless, direct oxidation of HQ on the bare electrode adsorbs the oxidized products over the electrodes’ surfaces, leading to the unstable response of the electrode and electrode fouling [31]. Additionally, one of the challenges resulting from overlapping their oxidation–reduction peak has been the direct detection of the dihydroxybenze isomers on the conventional electrodes such as GCEs [32,33].

It is well known that electrochemical performances can be tuned by tailoring the material compositions and surface properties of modified electrodes [34,35,36]. Moreover, analytical response due to the use of the modifiers is generally greater and thus the sensitivity of the electrode would largely improve. Hence, the well-organized modified electrodes must be immediately developed for detecting the analytes [37,38,39].

In recent years, the use of nanomaterials in various fields has progressed considerably [40,41,42,43,44,45,46,47,48]. On the one hand, scholars have utilized nanostructures as the modification materials because they have satisfactory features, including larger surface areas, and smaller sizes [49,50,51,52]. The design and fabrication of the nanomaterial-based electrochemical sensors by amplifying the signal and reducing overvoltage play an important role in electroanalysis. Graphene oxide (GO), one of the single layers of graphite oxide, was initially created by the graphite treatment with robust aqueous oxidizing agents. Studies have shown very good mechanical strength, higher mobility of the charge carriers, greater thermal conductivity, faster electron transport, and higher surface areas of the two-dimensional matter. These indicate its satisfactory features for possible uses in numerous areas [53,54,55]. Furthermore, it is possible to adjust graphene’s surface properties using chemical modifications, resulting in simple application in composite materials. Some studies have also referred to the synergetic effect of graphene and inorganic particles, resulting in the very good features and greater performance of the graphene-supported hybrids [56,57,58]. Manganese dioxide (MnO_2_), as one of the major functional materials, displays a higher surface area, inexpensiveness, stronger catalytic activities, as well as acceptable biocompatibility [59,60,61]. It should be noted that integrating the MnO_2_ nanoparticles (NPs) into the GO sheets potentially would increase the specific surface area and accelerate the transfer of electrons, leading to multiple channels and higher conductivity to diffuse the electrolyte ions [62,63]. So far, in some previous works, a GO/MnO_2_ nanocomposite with various morphologies of MnO_2_ has been used as an electrode material in the design and fabrication of electrochemical sensors for the determination of various analytes, and they showed good performance [22,64,65,66].

Herein, we developed the MnO_2_ NRs/GO/GCE for the sensitive detection of HQ in water samples. The results demonstrated that MnO_2_ NRs and GO had a good synergistic effect on the electrochemical oxidation of HQ. The other advantages of the sensor are excellent reproducibility, repeatability, stability, and high selectivity.

## 2. Materials and Methods

### 2.1. Instrumentation and Materials

According to the research design, we employed a General-Purpose Electrochemical System (GPES) 4.9 and Potentiostat/Galvanostat (Autolab PGSTAT302N, made in The Netherlands) for performing the electrochemical experiments.

All the electrochemical studies were performed at 25 ± 1 °C. A three electrode assembly was employed in the experiment in a 15 mL borosilicate glass cell containing GCE as a working electrode, which was bought from Azar electrode Co. (made in Urmia, Iran), a Pt wire as the counter electrode, and an Ag/AgCl (3 M KCl) reference electrode. The pH was also measured and buffer solution was prepared using a digital pH meter (Metrohm AG, Herisau, Switzerland, pH Lab 713). Deionized water used in each experiment was also taken from a Millipore Direct-Q^®^ 8 UV (ultra-violet) (Millipore, Germany). The morphological and elemental analyses of the prepared materials were carried out by a MIRA3 SEM (Tescan, Brno, Czech Republic).

It should be noted that the precursors for synthesizing the MnO_2_ NRs/GO nanocomposite, HQ, as well as other chemicals were also of analytical grade. It is noted that they were received from Merck and Sigma-Aldrich chemical companies. Furthermore, phosphate buffer solutions (0.1 M, PBS) of various levels of pH were provided via mixing the suitable contents of sodium hydroxide (NaOH) solution and phosphoric acid (H_3_PO_4_), also known as orthophosphoric acid under the pH-meter.

### 2.2. Synthesis of MnO_2_ NRs/GO Nanocomposite

For preparation of MnO_2_ NRs/GO nanocomposite, 20 mg GO powder was dispersed in deionized water (30 mL) under ultrasonic condition to form a homogeneous suspension. Afterwards, 0.316 gr of KMnO_4_ was poured into the GO suspension and 3.0 M HCl with strong stirring and continued stirring for half an hour. Next, this mixture was transported into the Teflon-lined autoclave at 160 °C for 6 h. Then, cooling was performed for collecting a black product through centrifugation and washed with ethanol and deionized water many times. After 12 h, an oven was used to dry MnO_2_ NRs/GO at 60 °C.

### 2.3. Modified Electrode Preparation

For the modification of GCE by using MnO_2_ NRs/GO nanocomposite, the drop-casting technique was employed. Then, 1 mg of the synthesized MnO_2_ NRs/GO was distributed in 1 mL of the deionized water in ultrasonication conditions for 30 min and the cast was provided by putting the 4 µL water/MnO_2_ NRs/GO nanocomposite suspensions on GCE and drying at ambient temperature.

The surface areas of the MnO_2_ NRs/GO/GCE and the bare electrode were obtained by CV using 1.0 mM K_3_Fe(CN)_6_ at several scan rates. By using the Randles–Sevcik equation, the value of the surface area for MnO_2_ NRs/GO/GCE (0.119 cm^2^) was 3.8 times greater than bare GCE.

### 2.4. Preparation of Water Specimens

River water and tap water were also sampled, filtrated with a membrane filter, and poured into 0.1 M PBS (pH = 7.0). At last, the HQ contents were measured in the water specimens using the as-developed protocol according to standard addition method.

## 3. Results and Discussions

### 3.1. Characterization of MnO_2_ NRs/GO Nanocomposite by Field Emission Scanning Electron Microscopy (FE-SEM)

**Figure 1:**
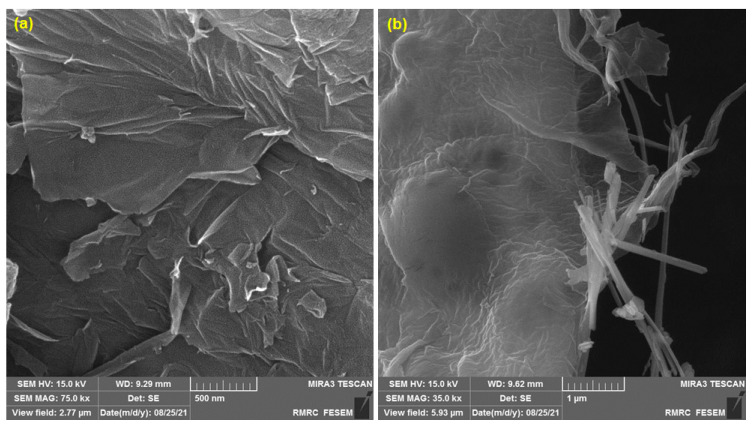
FE-SEM image of GO (**a**) and MnO_2_ NRs/GO nanocomposite (**b**).

FE-SEM analyses were conducted for studying the morphology and structural properties of the GO and MnO_2_ NRs/GO nanocomposite (Figure 1). The FE-SEM image of GO clearly shows that GO has a sheet structure (Figure 1a). In addition, the FE-SEM image of the nanocomposite (Figure 1b) represented the distribution and deposition of the MnO_2_ rod-shaped nanostructures on the GO sheets.

### 3.2. Electrochemical Behaviors of HQ on the MnO_2_ NRs/GO/GCE

**Figure 2:**
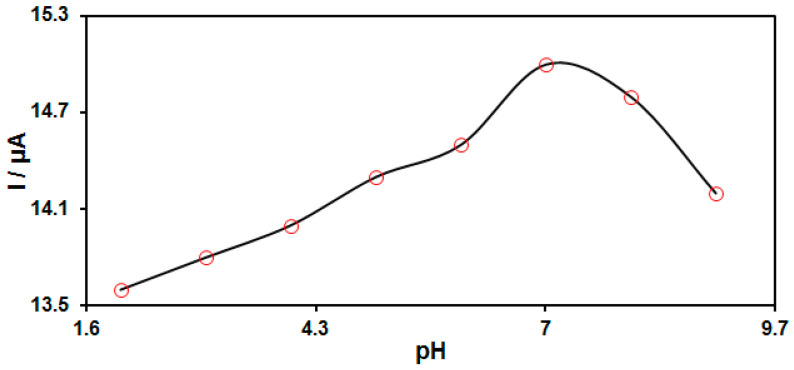
Plot of the oxidation peak current of 200.0 μM HQ as a function of pH solution on MnO_2_ NRs/GO/GCE in 0.1 M PBS at different pH value (2.0–9.0).

Studies revealed that electrochemical behaviors of HQ on the modified electrodes were followed by exchanges of protons. Therefore, the DPV technique was used for determining the effects of pH on the electrochemical response of MnO_2_ NRs/GO/GCE for HQ determination in various pHs of the PBS in ranges between 2.0 and 9.0, and concluded that a neutral medium would be more suitable to detect HQ electrochemically (Figure 2).

**Figure 3:**
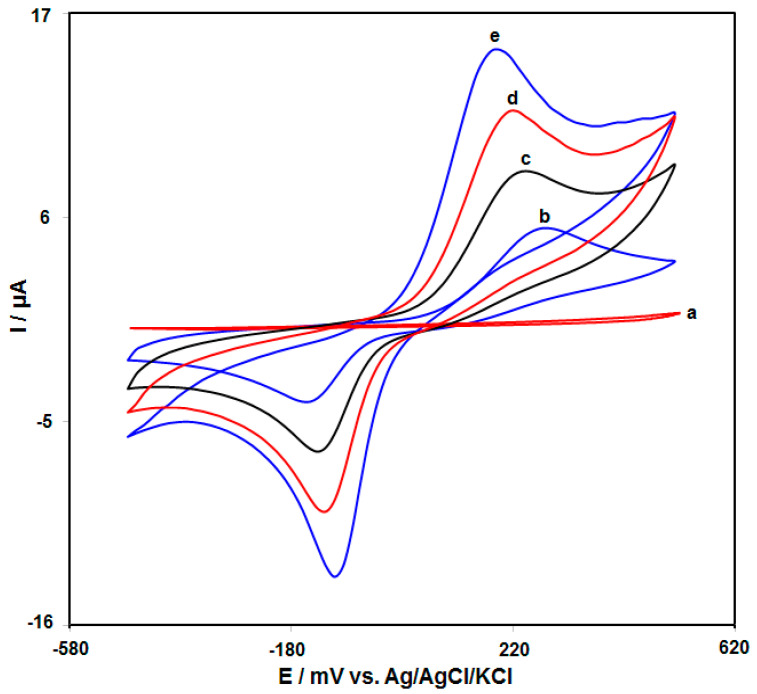
CVs of the (a) MnO_2_ NRs/GO/GCE in 0.1 M PBS (pH = 7.0) in the absence of HQ), (b–e) bare GCE, MnO_2_ NRs/GCE, GO/GCE and MnO_2_ NRs/GO/GCE in 0.1 M PBS (pH = 7.0) with 200.0 μM HQ. The scan rate was equal to 50 mV s^−1^.

Figure 3 presents the typical cyclic voltametric response of different electrodes in 0.1 M PBS (pH = 7.0) in the absence and presence of 200.0 μM HQ. According to Curve a, no peak was observed on the surface of modified electrode in the absence of HQ.

The results of electrochemical responses of 200.0 μM HQ in 0.1 M PBS (pH = 7.0) at the surface of bare GCE (b), MnO_2_ NRs/GCE (c), GO/GCE (d), and MnO_2_ NRs/GO/GCE (e) are shown in Table 1. Analysis indicated an enhancement in the cathodic and anodic peak currents by modifying the electrode surface and decrease of over potential. According to the results, MnO_2_ NRs/GO/GCE developed the greatest cathodic and anodic peak currents, demonstrating the maximum activities of the electrode surface for HQ redox reaction.

### 3.3. Scan Rate Exploration

**Figure 4:**
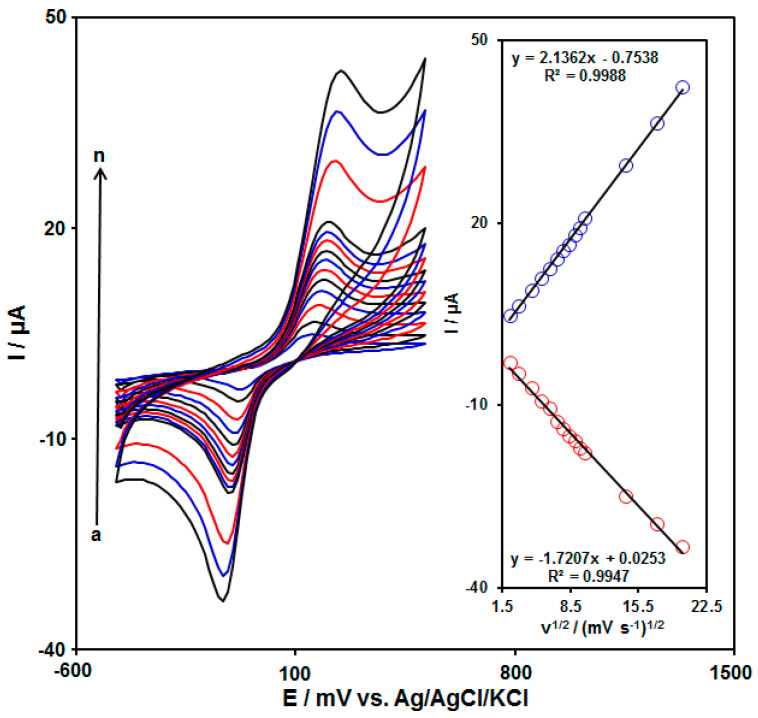
CVs of 120.0 μM HQ on MnO_2_ NRs/GO/GCE in 0.1 M PBS (pH = 7.0) at the various scanning rates (ν); (Curves a–n: (a) 5 mV/s, (b) 10 mV/s, (c) 20 mV/s, (d) 30 mV/s, (e) 40 mV/s, (f) 50 mV/s, (g) 60 mV/s, (h) 70 mV/s, (i) 80 mV/s, (j) 90 mV/s, (k) 100 mV/s, (l) 200 mV/s, (m) 300 mV/s, and (n) 400 mV/s. Inset: The plot of Ipa and Ipc vs. the square root of the scanning rate (υ^1/2^).

For studying the mechanisms required for the electrocatalytic redox reaction, the effects of the scanning rate (υ) on the reaction of 120.0 µM HQ on MnO_2_ NRs/GO/GCE ranged between 5 and 400 mVs^−1^. Considering Figure 4, as the scan rate enhanced, a linear increase in the cathodic and anodic peak currents (Ipc, Ipa) was observed. The inset of Figure 4 depicts the plots of the redox peak currents as a function of the square root of the scan rate (υ^1/2^) for HQ. The figure represents a linear increase in the redox peak currents with the square root of the scanning rates for different scan rates. Ultimately, analyses indicated the control of the electrode process through diffusion.

### 3.4. Chronoamperometric Determinations

**Figure 5:**
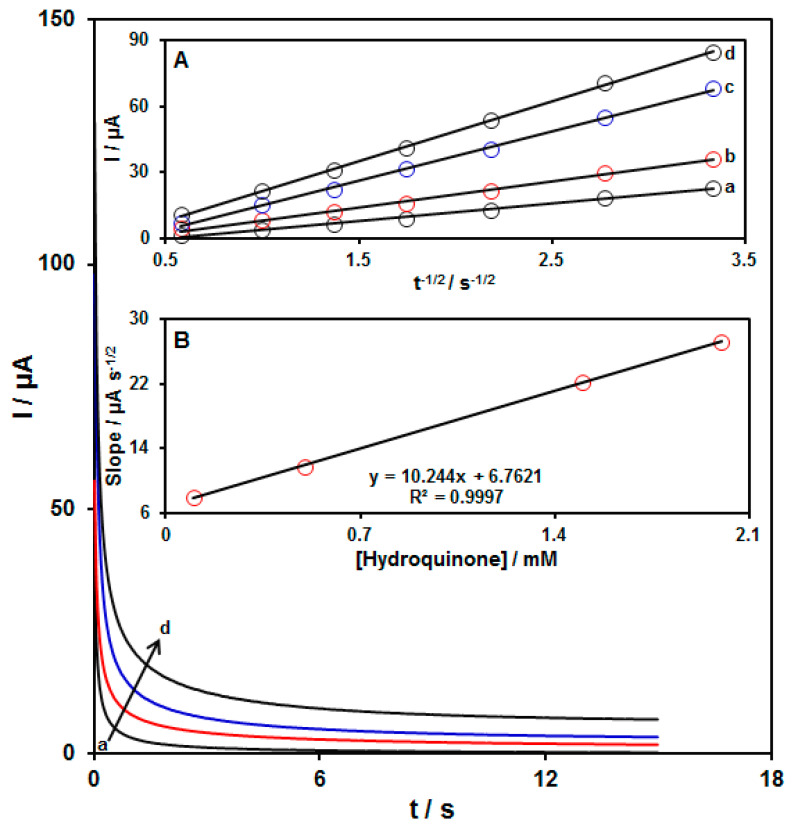
Chronoamperograms for the different concentrations of HQ on the MnO_2_ NRs/GO/GCE in 0.1 M PBS (pH = 7.0) in ranges between 0.1 and 2.0 mM (Curves a–d: (a) 0.1 mM, (b) 0.5 mM, (c) 1.5 mM, and (d) 2.0 mM). Insets: I-plots vs. t^−1/2^ for chronoamperograms a–d (**A**) and the slope from the straight lines vs. the concentration of HQ (**B**).

In this step, chronoamperometry was employed for HQ on the MnO_2_ NRs/GO/GCE surface (Figure 5) and the potential of the working electrode was set at 0.25 V for chronoamperometric measurements of various concentrations of HQ on the modified electrode surface. Then, through chronoamperometry, the diffusion coefficient (D) of HQ was specified in the aqueous solution by the Cottrell equation:

I = nFAC_b_D^1/2^π^−1/2^t^−1/2^

where C represents the known concentration, D stands for the apparent diffusion coefficient, and A refers to the electrode area. Experimental plots of I vs. t ^−1/2^ were also utilized for distinct concentrations of HQ (see Figure 5A) and slopes were drawn of the final straight lines versus the HQ concentration (Figure 5B). Finally, D was calculated according to the slope of the resulting plots with the Cottrell equation (8.9 × 10^−6^ cm^2^/s).

### 3.5. Calibration Plot

**Figure 6:**
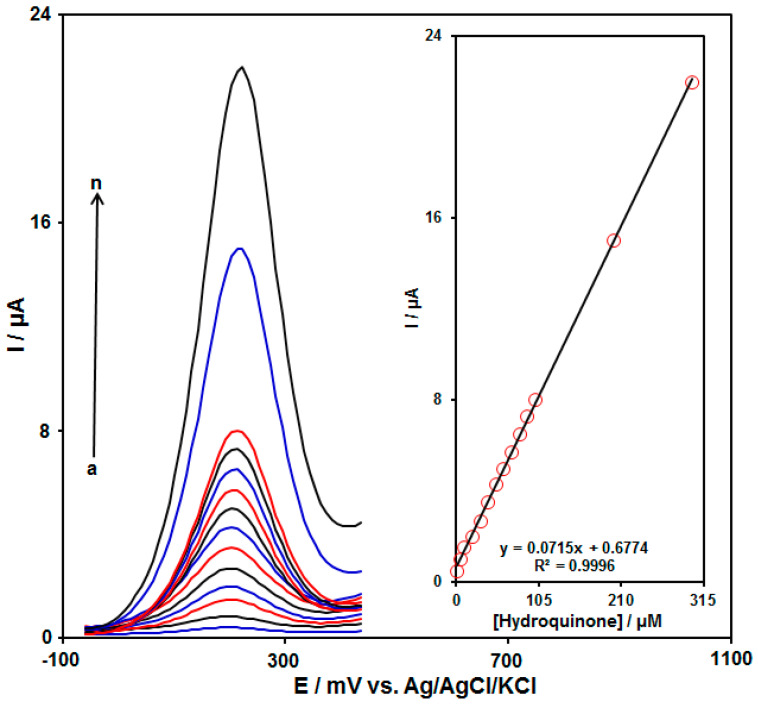
DPVs for diverse concentrations of HQ on MnO_2_ NRs/GO/GCE in 0.1 M PBS (pH = 7.0) in ranges from 0.5 to 300.0 μM (Curves a–n: (a) 0.5 μM, (b) 5.0 μM, (c) 10.0 μM, (d) 20.0 μM, (e) 30.0 μM, (f) 40.0 μM, (g) 50.0 μM, (h) 60.0 μM, (i) 70.0 μM, (j) 80.0 μM, (k) 90.0 μM, (l) 100.0 μM, (m) 200.0 μM, and (n) 300.0 μM). Inset: the related linear calibration curve of the peak current vs. concentration of HQ.

Differential pulse voltammetry (DPV) in the optimum experimental conditions was employed to quantitatively analyze the concentration of HQ in the PBS (0.1 M, pH = 7.0) on MnO_2_ NRs/GO/GCE. Figure 6 depicts the voltammograms for distinct concentrations of HQ. Considering the figure, as the HQ concentration enhanced, an increase in the peak currents was observed. Furthermore, the peak current linearly was related to the concentration of HQ in ranges between 0.5 and 300.0 µM (see Figure 6 (Inset)). For calibration curve of the modified electrode with an acceptable determination coefficient of 0.9996 and the lowest limit of detection of 0.012 µM, see the inset of Figure 6. The comparison of MnO_2_ NRs/GO/GCE sensor with other sensors for the determination of HQ is listed in Table 2. It can be seen that the MnO_2_ NRs/GO/GCE offers a proper linear range and a lower detection limit than some modified electrodes. Hence, our proposed method gives a straight and faster means of HQ detection in the samples.

### 3.6. Interference Studies

As a general principle, the relative error in the measurement is controlled at approximately ±5% and is considered to have no interference. To evaluate the selectivity of the fabricated MnO_2_ NRs/GO/GCE sensor, the influence of organic molecules and several common ions were assayed for 50.0 µM HQ. The results suggested that 120-fold of Pb^2+^, NH_4_^+^, Mg^2+^, Ca^2+^, NO_3_^−^, Na^+^, Cl^−^, K^+^, Al^3+^, and Fe^3+^ ions, 50-fold of citric acid, vitamin B_12_, CH_3_COOH and 5-fold excess of uric acid did not interfere with the determination of HQ (the relative errors were within ±5%). However, catechol, dopamine, and ascorbic acid in equal molar concentrations showed interference.

### 3.7. Stability, Repeatability, and Reproducibility Studies

In order to determine the stability of the MnO_2_ NRs/GO/GCE, its electrocatalytic response to 50.0 μM HQ in 0.1 M PBS was monitored every day (1 to 14 day intervals). The electrode presented consistent voltametric responses to HQ during the 14-day storage period. About 96.1% of initial response current was maintained after 14 days of its consecutive use and this result denoted the admissible stability of the proposed modified electrode (MnO_2_ NRs/GO/GCE).

Repeatability and reproducibility of the proposed MnO_2_ NRs/GO-based sensor have been evaluated using voltametric studies in 0.1 M PBS (pH = 7.0) containing 50.0 µM HQ. The MnO_2_ NRs/GO/GCE sensor presented appreciable repeatability with relative standard deviation (R.S.D) of 4.9% for 6 repetitive measurements performed using an individua electrode.

To check the reproducibility, the five MnO_2_ NRs/GO/GCE were applied in the determination of HQ. Experiments with 3.3% (%RSD) for 50 µM HQ in 0.1 M PBS (pH = 7.0) were done, which showed the acceptable reproducibility of the fabricated sensor for determine HQ.

### 3.8. Application of the MnO_2_ NRs/GO/GCE Sensor for the Determination of HQ in Real Samples (River Water and Tap Water)

The capability of the electrocatalytic oxidation of HQ in the real samples was studied by voltammetry in the water samples (river water and tap water). Table 3 reports the results. As seen, the method recoveries ranged between 97.3 and 101.4%, reflecting the capability of MnO_2_ NRs/GO/GCE for voltametric detection of HQ with acceptable reproducibility.

## 4. Conclusions

In this study, a sensitive electrochemical sensor was made based on the MnO_2_ NRs/GO/GCE to detect HQ. It was produced through a simple drop-casting of the MnO_2_ NRs/GO nanocomposite dispersion over the GCE surface. As the synergistic effects of GO and MnO_2_ NRs were applied, HQ’s redox peak currents largely enhanced in comparison to the bare GCE. According to the findings, MnO_2_ NRs/GO/GCE had very good sensing functions to determine HQ, with a linear range (0.5 µM–300.0 µM) and lower LOD of 0.012 µM. Finally, the MnO_2_ NRs/GO/GCE could be satisfactorily utilized for HQ detection in the real samples. The other advantages of the sensors are excellent reproducibility, repeatability, stability, and high selectivity.

## Figures and Tables

**Table 1:** Comparison the electrochemical responses of 200.0 μM HQ in 0.1 M PBS (pH = 7.0) at the surface of different electrodes.

Electrode	Anodic Peak Current (µA)	Anodic Peak Potential (mV)	Cathodic Peak Current (µA)	Cathodic Peak Potential (mV)
Bare GCE	5.4	290	−4.0	−140
MnO_2_ NRs/GCE	8.5	240	−6.5	−130
GO/GCE	11.7	215	−9.9	−115
MnO_2_ NRs/GO/GCE	15.0	200	−13.4	−100

**Table 2:** Comparison the sensing performances toward the detection of HQ between the existing electrochemical sensors and the proposed MnO_2_ NRs/GO/GCE sensor.

Electrochemical Sensor	Analytical Methods	Dynamic Linear Range	Limit of Detection	Ref.
Zinc @ zinc oxide core-shell/glassy carbon electrode	Cyclic voltammetry	10.0 to 90.0 µM	0.10443 µM	[14]
Glassy carbon electrode modified with multiwall carbon nanotubes	Differential pulse voltammetry	1.0 × 10^−6^ M to 1.0 × 10^−4^ M	7.5 × 10^−7^ M	[15]
Reduced graphene oxide cross-linked L-cysteine/glassy carbon electrode	Differential pulse voltammetry	2.0 to 160.0 µM	1.5 µM	[16]
Au@Pd nanocomposites/glassy carbon electrode	Differential pulse voltammetry	4.0 to 5000.0 µM	0.63 µM	[17]
Poly-amidosulfonic acid and multi-wall carbon nanotubes composite electropolymerization on glassy carbon electrode	Differential pulse voltammetry	6.0 × 10^−6^ to 4.0 × 10^−4^ M	1.0 × 10^−6^ M	[18]
Nanodiamond/glassy carbon electrode	Differential pulse voltammetry	1.0 to 78.0 μM	0.19 μM	[19]
CuS nanocrystals/chitosan/glassy carbon electrode	Cyclic voltammetry	4.5 µM to 4.5 mM	1.5 µM	[20]
Electrodeposition of reduced graphene oxide on glassy carbon electrode	Differential pulse voltammetry	6.0 to 200.0 μM	0.2 μM	[21]
GO–mesoporous MnO_2_ nanocomposite/glassy carbon electrode	Differential pulse voltammetry	0.01 to 0.7 µM	7.0 nM	[22]
MnO_2_ NRs/GO/GCE	Differential pulse voltammetry	0.5 to 300.0 μM	0.012 µM	This Work

**Table 3:** HQ detection in the real samples with the MnO_2_ NRs/GO/GCE (concentration in µM (*n* = 5)).

Sample	Spiked	Found	Recovery (%)	R.S.D. (%)
River water	0	-	-	-
	5.0	4.9	98.0	3.5
	7.0	7.1	101.4	1.9
Tap Water	0	-	-	-
	5.5	5.7	103.6	2.8
	7.5	7.3	97.3	2.1

## Data Availability

The data presented in this study are available on request from the corresponding authors.
